# Mucosal Vaccination Primes NK Cell-Dependent Development of CD8^+^ T Cells Against Pulmonary *Brucella* Infection

**DOI:** 10.3389/fimmu.2021.697953

**Published:** 2021-07-07

**Authors:** Ella Bhagyaraj, Hongbin Wang, Xinghong Yang, Carol Hoffman, Ali Akgul, Zakia I. Goodwin, David W. Pascual

**Affiliations:** Department of Infectious Diseases & Immunology, University of Florida, Gainesville, FL, United States

**Keywords:** *Brucella*, dendritic cells, macrophages, IFN-γ, chemokines, CD8^+^ T cells

## Abstract

Past studies with the live, double-mutant *B. abortus* (znBAZ) strain resulted in nearly complete protection of mice against pulmonary challenge with wild-type (wt) *Brucella via* a dominant CD8^+^ T cell response. To understand the contribution innate immune cells in priming CD8^+^ T cell responses, mice were nasally dosed with wt *B. abortus*, smooth vaccine strain 19 (S19), or znBAZ, and examined for innate immune cell activation. Flow cytometric analysis revealed that znBAZ, but not wt *B. abortus* nor S19 infection, induces up to a 5-fold increase in the frequency of IFN-γ-producing NK cells in mouse lungs. These NK cells express increased CXCR3 and Ki67, indicating their recruitment and proliferation subsequent to znBAZ infection. Their activation status was augmented noted by the increased NKp46 and granzyme B, but decreased NKG2A expression. Further analysis demonstrated that both lung caspase-1^+^ inflammatory monocytes and monocyte-derived macrophages secrete chemokines and cytokines responsible for NK cell recruitment and activation. Moreover, neutralizing IL-18, an NK cell-activating cytokine, reduced the znBAZ-induced early NK cell response. NK cell depletion also significantly impaired lung dendritic cell (DC) activation and migration to the lower respiratory lymph nodes (LRLNs). Both lung DC activation and migration to LRLNs were significantly impaired in NK cell-depleted or IFN-γ^-/-^ mice, particularly the CD11b^+^ and monocytic DC subsets. Furthermore, znBAZ vaccination significantly induced CD8^+^ T cells, and upon *in vivo* NK cell depletion, CD8^+^ T cells were reduced 3-fold compared to isotype-treated mice. In summary, these data show that znBAZ induces lung IFN-γ^+^ NK cells, which plays a critical role in influencing lung DC activation, migration, and promoting protective CD8^+^ T cell development.

## Introduction

Brucellosis is one of the most prevalent bacterial zoonotic diseases worldwide and listed by the World Health Organization as one of the “seven most neglected diseases” (1–3). *Brucella*, a Gram-negative, facultative bacterium, is responsible for this disease. While commonly thought of as a disease of livestock, the four closely-related *Brucella* species – *B. abortus*, *B. melitensis*, *B. suis*, and *B. canis -* can cause human disease (4, 5). *Brucella* is transmitted from infected animals to humans through direct contact, ingestion of contaminated foods, or by inhalation of *Brucella*-laden aerosols (6). The animal and human forms of brucellosis tend to differ symptomatically – in livestock, brucellosis causes abortion, infertility, mastitis, and lameness, whereas in humans, the effects are incapacitating illness characterized by undulating fever with flu-like symptoms, which can persist if untreated (5, 7, 8). An estimate of 500,000 humans are annually infected with *Brucella* (9, 10). Moreover, chronic brucellosis can lead to additional complications in humans such as endocarditis, arthritis, epididymo-orchitis, and sacroiliitis (11–14). The most common routes of infection in humans are oral *via* consumption of contaminated foods or inhalation (15). Thus, induction of strong mucosal immunity in the aerodigestive tracts is desirable to provide efficient and long-lasting protection against *Brucella* infection. Given the routes of exposure, a mucosal vaccine that activates immunity in the lungs and gut seems a logical step to advancing *Brucella* vaccines. Mucosal vaccination is advantageous because antigen-specific humoral and cell-mediated immune responses can be induced both in the mucosal and systemic compartments (16–18). As such, mucosal vaccines may provide better coverage against pathogens than those given parenterally.

Embedded within the mucosal tissues are innate immune cells, which play a crucial role in antimicrobial defenses. Stimulation of innate immunity by respiratory pathogens is central to generating a pathogen-specific immunity (19, 20). Among the innate immune cell population in the lungs, NK cells merit special attention due to their role as a bridge between innate and adaptive immune responses (21, 22). The lungs harbor NK cells, underscoring their importance in respiratory mucosal immune protection (23). NK cells act as an essential first line of defense against respiratory infections, and are poised to exert effector functions and produce cytokines and chemokines that coordinate innate and adaptive immune responses (24, 25). Early in infection, NK cells are primarily activated by cytokines such as type I interferons, interleukin-12 (IL-12), IL-15, and IL-18, secreted by macrophages and monocytes (26, 27). NK cells activation is also regulated by a wide array of soluble or membrane bound ligands on infected cells that interact with both activating and inhibitory receptors on the NK cell surface (28). Activated NK cells protect against invasive respiratory pathogens either by direct lysis of infected cells or indirectly by activating other innate immune cells such as macrophages or dendritic cells (DCs) (29). In addition, NK cells can indirectly modulate adaptive immune responses by its assistance in priming T cell responses (30).

The present study describes a live, double-mutant Δ*norD* Δ*znuA* *Brucella abortus* (znBAZ) strain that, after mucosal vaccination, confers complete protection against pulmonary challenge with virulent *B. abortus* 2308 by stimulating robust CD8^+^ T cell responses in the lungs (31). To better understand how znBAZ confers protection, the role of innate cells, particularly, NK cells was examined. A robust, early activation of NK cells was induced following nasal znBAZ infection, but not observed when infected with wild-type (wt) *B. abortus* 2308 or *B. abortus* S19 vaccine. Additional mechanistic studies were conducted to discern the impact of NK cells has upon znBAZ-induced lung CD8^+^ T cell responses, and the relevance of IFN-γ in innate cell activation and migration. These results provide important insights regarding the role of early lung NK cell activation in znBAZ-mediated immunity.

## Materials and Methods

### Bacterial Strains and Culture Conditions

The construction of znBAZ, a live attenuated mutant *Brucella abortus* strain, was previously described, and lacks functional *znuA* and *norD* genes (32). All *B. abortus* vaccine strains, S19, RB51, znBAZ, and wt 2308, were inoculated and grown on Potato Infusion Agar (PIA) plates for three days at 37°C under 5% CO2. Before infection, bacteria were harvested, washed, and diluted in sterile phosphate-buffered saline (sPBS) for use.

### Mice

BALB/c and C57BL/6 (female, 6–8 weeks old) mice were obtained from Charles River Laboratory (Frederick, MD, USA), and IFN‐γ^−/−^ mice on C57BL/6 background were bred in-house. All animal experiments performed with live attenuated *Brucella* vaccine strains S19 and znBAZ were conducted under biosafety level-2 (BSL-2) containment; studies involving wt *B. abortus* 2308 were done under BSL-3 containment. Mice were maintained in individually ventilated cages under HEPA-filtered barrier conditions with 12 h of light and 12 h of darkness, and food and water were provided *ad libitum*. All animal care and procedures were in strict accordance with the recommendations in the Guide for the Care and Use of Laboratory Animals of the National Institutes of Health. All animal studies were conducted under protocols approved by the University of Florida Institutional Animal Care and Use Committee.

### Infection

BALB/c or C57BL/6 mice (n = 5/group) were nasally dosed with 30 μl of sPBS, 1×10^5^ CFUs wt *B. abortus* 2308, 1×10^8^ CFUs S19, or 1×10^9^ CFUs znBAZ administered into the anterior nares dropwise using a micropipette under isoflurane anesthesia. The infected mouse lungs and lower respiratory lymph nodes (LRLNs) were harvested at different time points (2, 5, and 15 days) after infection and analyzed for brucellae colonization and mononuclear cell composition. Lung and splenic homogenates from individual mice were plated on Farrell’s medium (Oxoid Ltd, Basingstoke, UK) or PIA for 3–5 days at 37°C in 5% CO_2_.

### Bronchoalveolar Lavage Fluid

Nasally infected and uninfected mice were euthanized, their tracheas were cannulated, and the lungs were perfused with 1 ml of cold sPBS three times, and a total of 3 ml BAL fluids were collected from each mouse. BAL fluid samples were centrifuged at 500 × g for 10 min at 4°C, and recovered fluids were filter-sterilized using 0.22 µ syringe filters. The sterile BAL fluid samples were stored at −70°C for cytokine analysis.

### Cytokine ELISAs

Cytokines (IFN-γ, IL-12p70, IL-15, IL-18) and chemokines (CXCL-9 and CXCL-10) levels were measured from BAL fluids (duplicate samples) collected from mice dosed nasally with wt *B. abortus* 2308, S19, or znBAZ, along with those from uninfected mice by capture ELISA using the following antibody pairs: IFN-γ (clone R4-6A2 and clone XMG1.2 from BD Pharmingen), IL-12p70 (clone 9A5 and biotinylated clone C17.8; BD Pharmingen), IL-15 (clone 201136 and biotinylated polyclonal goat IgG anti-mouse IL-15; R&D Systems), IL-18 (clone 74 and biotinylated clone 93-10C; R&D Systems), CXCL-9 (polyclonal goat and biotinylated polyclonal goat IgG anti-mouse CXCL-9; R&D Systems), and CXCL-10 (clone 134013 and biotinylated polyclonal goat IgG anti-mouse CXCL-10; R&D Systems). ELISAs were developed using a third step antibody Ab, horseradish peroxidase (HRP) conjugated goat anti-biotin Ab (Vector Laboratories). After a wash step, ABTS peroxidase substrate (Moss, Inc., Pasadena, ME, USA) was added to each well, and reactions were read at 415 nm using a Bio-Tek Instruments Epoch Microplate Spectrophotometer (Winooski, VT). Cytokine concentrations were extrapolated from standard curves generated by recombinant murine cytokines and chemokines: IFN-γ (Peprotech), IL-15 (R&D Systems), IL-12 (R&D Systems), IL-18 (R&D Systems), CXCL-9 (R&D Systems), and CXCL-10 (R&D Systems). ELISA methods used were similar to those previously described (31, 32).

### Single-Cell Preparation and *In Vitro* Stimulation

LRLNs and lungs were aseptically removed from euthanized mice and collected into 2ml tubes containing 1ml of incomplete media (ICM): RPMI-1640, 10 mM HEPES buffer, and 10 mM penicillin/streptomycin. These tissues were then mechanically homogenized and filtered through 70-µm cell strainers (Fisherbrand) to obtain single-cell suspensions. Lung tissue was additionally digested with 20 μg of Liberase TL research-grade (Roche) and 50 units of RNase-free DNase I (Promega) for 45 min at 37°C under 5% CO2, followed by 0.5M EDTA treatment for 5 mins. Cells were then suspended in freshly made ammonium-chloride-potassium (ACK) lysis buffer for 3 mins, then washed with ICM, and resuspended in complete media (CM): ICM plus 10% fetal bovine serum (FBS), 10 mM nonessential amino acids, and 10 mM sodium pyruvate. For flow cytometry analysis, single-cell suspensions were either stimulated overnight with heat-killed RB51 (HKRB51) followed by 4 hours of 5 ng/ml phorbol myristate acetate (PMA; SIGMA-ALDRICH), 500 ng/ml ionomycin (SIGMA-ALDRICH) and 10 μg/ml brefeldin A (SIGMA-ALDRICH) before antibody (Ab) staining or stained directly.

### Flow Cytometry Assay

For flow cytometry analysis, cells were washed with PBS plus 2% FBS, and incubated with Fc blocker (eBioscience) along with Live/dead stain (ThermoFisher) for 15 min at 4°C. After an additional wash, the cells were surface stained for 30 min at 4°C with mAbs specific for CD49b (DX5), NK1.1 (PK136), CD4 (GK1.5), CD8 (53-6.7), CCR2 (SA203G11), CCR5 (HM-CCR5), CXCR3 (CXCR3-173), NKG2D (CX5), NKp46 (29A1.4), NKG2A (16A11), and TCR-β (H57-597) for lymphocyte analysis. For macrophages and dendritic cells, cells were stained with mAbs specific for SiglecF (1RNM44N), CD11b (M1/70), CD11c (N418), CD103 (2E7), F4/80 (BM8), Ly6C (HK1.4), Ly6G (1A8), CD40 (3/23), CD80 (16-10A1), CD86 (GL-1), and MHCII (M5/114.15.2). For detection of asialo-GM1^+^ T cells, the rabbit polyclonal anti-asialo-GM-1 Ab was used (eBioscience). For intracellular staining, the surface stained cells were washed, fixed, and permeabilized using the True-Nuclear Transcription Factor Buffer Set (BioLegend, San Diego, CA) followed by intracellular staining with mAbs specific for IL-6 (MP5-20F3), IL-12 (C15.6), IL-18 (93-10C), IFN-γ (XMG1.2), TNF-α (MP6-XT22), Ki-67 (SoIA15), iNOS (CXNFT), and granzyme-B (NGZB). All the fluorescently conjugated mAbs were procured from (BioLegend, San Diego USA) or (eBioscience, San Diego USA). After staining, cells were acquired on a BD Fortessa flow cytometer and analyzed by using FlowJo software.

### Active Caspase-1 Detection

Activated caspase-1 was detected in cells by using FLICA assay (Immunochemistry Technology) as per manufacturer guidelines. Briefly, cells were incubated with FLICA reagent (FAM-YVAD-FMK) for 30 mins at 37°C. These FLICA stained cells were then washed with PBS containing 2% FBS and subjected to surface staining before flow cytometry analysis.

### Anti-IL-18 mAb Treatment

IL-18 was neutralized *in vivo* using 200 µg/dose/mouse of anti-mouse IL-18 mAb (clone YIGIF74-1G7; Bio X Cell). Mice were treated i.p with isotype control IgG (Bio X Cell) or anti-mouse IL-18 mAb on day -1 (one day before infection) and every three days after that until the termination of the experiment. Mice were infected with 1×10^9^ CFUs znBAZ nasally on day 0.

### *In Situ* Carboxy-Fluorescein Succinimidyl Ester Staining and Lung DC Migration

Lung DC migration was monitored by using *in situ* CFSE staining (33). 25mM CFSE (Molecular Probes) was diluted in sPBS to a concentration of 5mM. Mice were anesthetized, and nasally instilled with 30 µl of diluted CFSE for labeling lung cells *in vivo* 6 hr before nasal infection. Five days after infection, the LRLNs were analyzed for CFSE^+^ DC subsets.

### NK Cell Depletion Studies

NK cells were depleted using 50 µg/dose/mouse of rabbit anti-asialo-GM1 antibody (eBioscience) in BALB/c mice or anti-NK1.1 (clone PK136; Bio X Cell) in C57BL/6 mice. Mice were treated i.p with isotype control IgG or anti-asialo-GM1 Ab or anti-NK1.1 on day -1 (one day before infection) and every three days after until the termination of the experiment. Mice were nasally infected with 1×10^9^ CFUs znBAZ on day 0. NK cell depletion in the lungs and spleens was confirmed by flow cytometry.

### Statistical Analysis

All experiments were conducted two or three times with n=5 mice/group. The statistical significance of the data was calculated by using One-way ANOVA followed by Tukey’s multiple comparison test with SigmaPlot 12.0. All results were discerned to the 95% confidence interval.

## Results

### Nasal znBAZ Infection Elicits an Early NK Cell Response in Mouse Lungs

**Figure 1:**
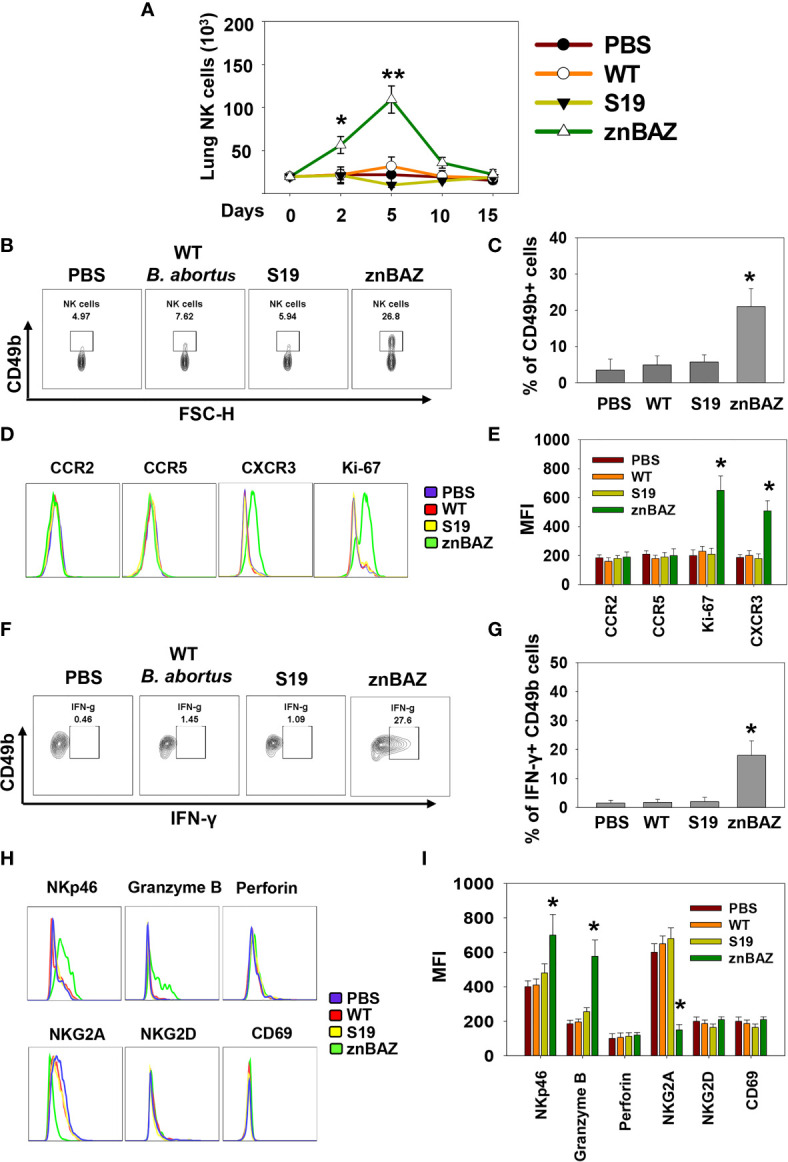
Nasal znBAZ infection promotes lung NK cell expansion. Groups of BALB/c mice were nasally infected with wild-type (wt) *B. abortus* 2308 (1×10^5^ CFUs), *B. abortus* S19 vaccine (1×10^8^ CFUs), or znBAZ (1×10^9^ CFUs). **(A)** Lung NK cell numbers were measured at the indicated days post-infection. **(B, C)** Flow cytometry analysis of NK (gated on TCRβ^-^ CD49b^+^) cells on day 5 post-infection, and examined for **(D, E)** CCR2, CCR5, CXCR3 and Ki67 expression. **(F, G)** On day 3 post-infection, expression of IFN-γ-producing lung NK cells is depicted. **(H)** Lung NK cells for (top row) NKp46, granzyme B, perforin, (bottom row), NKG2A, inhibitory molecule, NKG2D, and CD69, and their respective **(I)** MFIs are shown. The data depict the means ± SEM of 5 mice/group; *p < 0.01 and **p ≤ 0.001, compared with PBS-dosed mice. Data are representative of two experiments.

To determine the role of NK cells in establishing znBAZ-induced protective immunity in the lungs, groups of BALB/c mice were nasally dosed with wt *B. abortus* 2308, *B. abortus* S19 vaccine, or znBAZ. Different doses were used since wt *B. abortus* 2308 and S19 are pathogenic in mice, whereas znBAZ is a highly attenuated strain (31) compared to these strains. znBAZ showed a modest increase in lung colonization by day 10, but afterwards declined by greater than two-logs by day 15 (**Supplementary Figure 1A**). S19 retained elevated levels over the entire course, and showed no indication of decline. Wt *B. abortus* 2308 achieved similar levels as S19 by day 10, and continued to increase exceeding znBAZ colonization by greater than two logs (**Supplementary Figure 1A**). The S19 vaccine was selected since it is a smooth vaccine resembling znBAZ. Lung lymphocyte populations were analyzed at different time points to assess when NK cells (gating strategy provided in **Supplementary Figure 1B**) became involved. Subsequent to znBAZ infection, the frequency of lung NK cells gradually increased and peaked after 5 days, in contrast to lung NK cell levels in mice infected with wt *B. abortus* or S19 vaccine resembled uninfected mice (**Figures 1A–C**). The early surge in lung NK cells was due to either their recruitment or proliferation of sentinel NK cells. To distinguish between the two possibilities, the expression of the proliferation marker Ki67, along with chemokine receptors, CCR2, CCR5, and CXCR3, which aid with NK cell recruitment, was analyzed on lung NK cells 3 days after nasal instillation. A significant increase in Ki67 and CXCR3 expression by NK cells was evident, but CCR2 and CCR5 levels remained unchanged, suggesting that NK cell infiltration and proliferation occurred after znBAZ infection (**Figures 1D, E**). Additionally, NK cells serve as an important early sentry and a source of IFN-γ, crucial to initiating the adaptive immune response following infection. At day 5 post-infection, the lung NK cells showed significantly enhanced IFN-γ in znBAZ-infected mice (**Figures 1F, G**). IFN-γ expression by NK cells from wt *B. abortus*- or S19-infected mice did not vary from naïve levels. Hence, the escalation in NK cells was not evident in the lungs from wt *B. abortus*- or S19-infected mice.

Lung NK cells from znBAZ-infected mice displayed an activated status unlike those from mice infected with wt *B. abortus* or S19. Levels of granzyme-B and NK cell activation molecule, NKp46, increased significantly in znBAZ-dosed mice, in contrast to wt *B. abortus* or S19. Notably, expression of NK cell inhibitory molecule, NKG2A, was unchanged in wt *B. abortus*- and S19-infected mice relative to naïve controls, but NKG2a was significantly reduced in znBAZ-infected mice (**Figures 1H, I**). These findings were validated in C57BL/6 mice, showing similar results (**Supplementary Figures 1C–H**). Collectively, these findings support the notion that nasal znBAZ infection activates NK cells, while nasal infection with wt *B. abortus* or S19 does not alter lung NK cell numbers nor their activation status.

### Nasal Infection With znBAZ Elicits NK Cell-Activating Cytokines and Chemokines to Recruit Lung Monocytes and Macrophages

**Figure 2:**
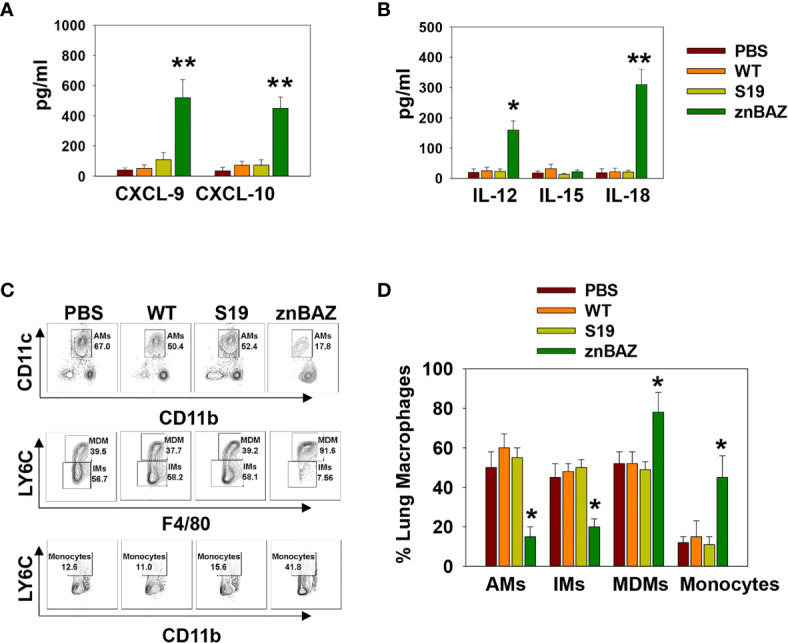
Lung monocytes and macrophages produce CXCR3 ligands, CXCL-9 and CXCL-10, and NK cell-activating cytokines, IL-12 and IL-18, subsequent to nasal znBAZ infection. Groups of BALB/c mice were nasally dosed with wild-type *B. abortus* 2308 (WT), *B. abortus* S19 vaccine, or znBAZ, and three days later, **(A)** CXCR3 ligands, CXCL-9 and CXCL-10, and **(B)** NK cell activating cytokines, IL-12, IL-15, and IL-18, present in individual bronchoalveolar lavage (BAL) fluids, were measured by cytokine-specific ELISA. **(C, D)** Total mononuclear cells were isolated from the lungs to measure the frequency of lung monocytes and macrophages including alveolar macrophages (AMs), interstitial macrophages (IMs), monocyte-derived macrophages (MDMs), and monocytes by flow cytometry. The data depict the means ± SEM of 5 mice/group; *p < 0.01 and **p ≤ 0.001, compared with sPBS-dosed mice. Data are representative of two or three experiments.

To identify the cytokines and chemokines that contribute to early lung NK cell activation upon nasal znBAZ infection, groups of BALB/c mice were nasally infected with wt *B. abortus*, S19, or znBAZ, and 3 days later, individual BALs and lungs were harvested to measure chemokine and cytokine levels produced locally. Upon znBAZ infection, CXCR3 ligands, CXCL-9, and CXCL-10 levels were significantly enhanced by 14- and 12-fold, respectively (**Figure 2A**). Likewise, NK cell-activating cytokines, IL-12 and IL-18, were notably elevated by 4- and 10-fold, respectively (**Figure 2B**). However, wt *B. abortus* and S19 showed no significant difference in CXCL-9, CXCL10, IL-12, IL-15, or IL-18 production compared to PBS-dosed mice (**Figures 2A, B**).

**Figure 3:**
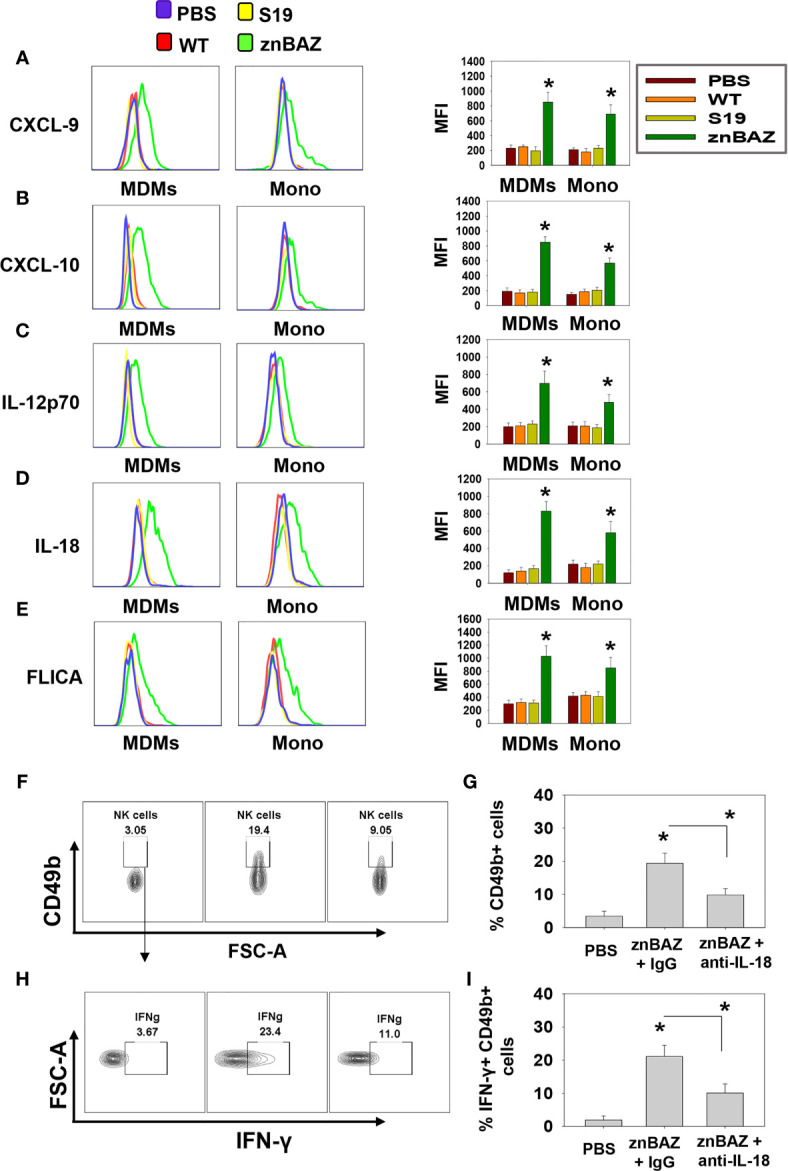
The source of CXCL-9, CXCL-10, IL-12, and IL-18 are from monocyte-derived macrophages (MDMs) and monocytes (Mono). **(A–E)** Flow cytometry analysis of lung MDMs and monocytes was performed three days after infection to determine the source and mean fluorescence intensity (MFI) of CXCL-9, CXCL-10, IL-12p70, IL-18, and activation of caspase-1 (FLICA-positive) cells. Flow cytometry analysis of lung **(F, G)** CD49b^+^ and **(H, I)** IFN-γ-producing CD49b^+^ NK cells on day 5 post-znBAZ infection of BALB/c mice i.p treated with anti-IL-18 mAb or an equivalent amount IgG isotype control on days -1 and +2. The data depict the means ± SEM of 5 mice/group; *p < 0.01 compared with PBS-dosed mice or as indicated. Data are representative of two or three experiments.

To determine the cell source of these chemokines and cytokines, flow cytometry analysis was performed on stained lung mononuclear cells isolated from uninfected and infected mice 3 days after nasal infection. The gating strategy for identifying myeloid lung cells is presented in **Supplementary Figure 2**. SiglecF^+^ CD11b^lo^ CD11c^+^ alveolar macrophages (AMs) were notably reduced, as were SiglecF^-^ CD11b^+^ Ly6G^-^ F4/80^+^ Ly6C^-^ interstitial macrophages (IMs; **Figures 2C, D**). In contrast, the SiglecF^-^ CD11b^+^ Ly6G^-^, F4/80^+^, Ly6C^+^ monocyte-derived macrophages (MDMs) and SiglecF^-^ CD11b^+^ Ly6G^-^ F4/80^-^ Ly6C^+^ lung monocytes were significantly increased by 1.5- and 3-fold, respectively, in the znBAZ-infected mice. The macrophage and monocyte subsets remained unchanged in the lungs from those infected with wt *B. abortus* 2308 or S19 (**Figures 2C, D**). Importantly, the lung MDMs and monocytes from the znBAZ-infected mice produced elevated CXCL-9, CXCL-10, IL-12, and IL-18 (**Figures 3A–D**). Caspase-1 is necessary to activate pro-IL-18 (34, 35), so as expected, the caspase-1 activity was found associated with these mononuclear cells (**Figure 3E**). During infections, APC-elicited IL-18 stimulates IFN-γ production by NK cells (36). IL-18 often works synergistically with IL-12 resulting in elevated IFN-γ production by NK cells, and actually, IL-18 alone is sufficient to enhance IFN-γ production (37, 38). Given the importance of IL-18 in NK cell activation and its elevated presence following nasal znBAZ infection, the role of IL-18 was investigated. Groups of BALB/c mice were treated by the i.p. route with either anti-IL-18 mAb or IgG isotype control one day prior to nasal znBAZ infection and every three days afterwards. Subsequent to *in vivo* IL-18 neutralization, analysis of lung lymphocytes at day 5 post-infection revealed a significant decline in the frequency of total NK cells and IFN-γ^+^ NK cells by 2.2-fold for both (**Figures 3F–I**).

### znBAZ-Induced Lung NK Cell Response Is Essential for Lung DC Maturation and Migration

**Figure 4:**
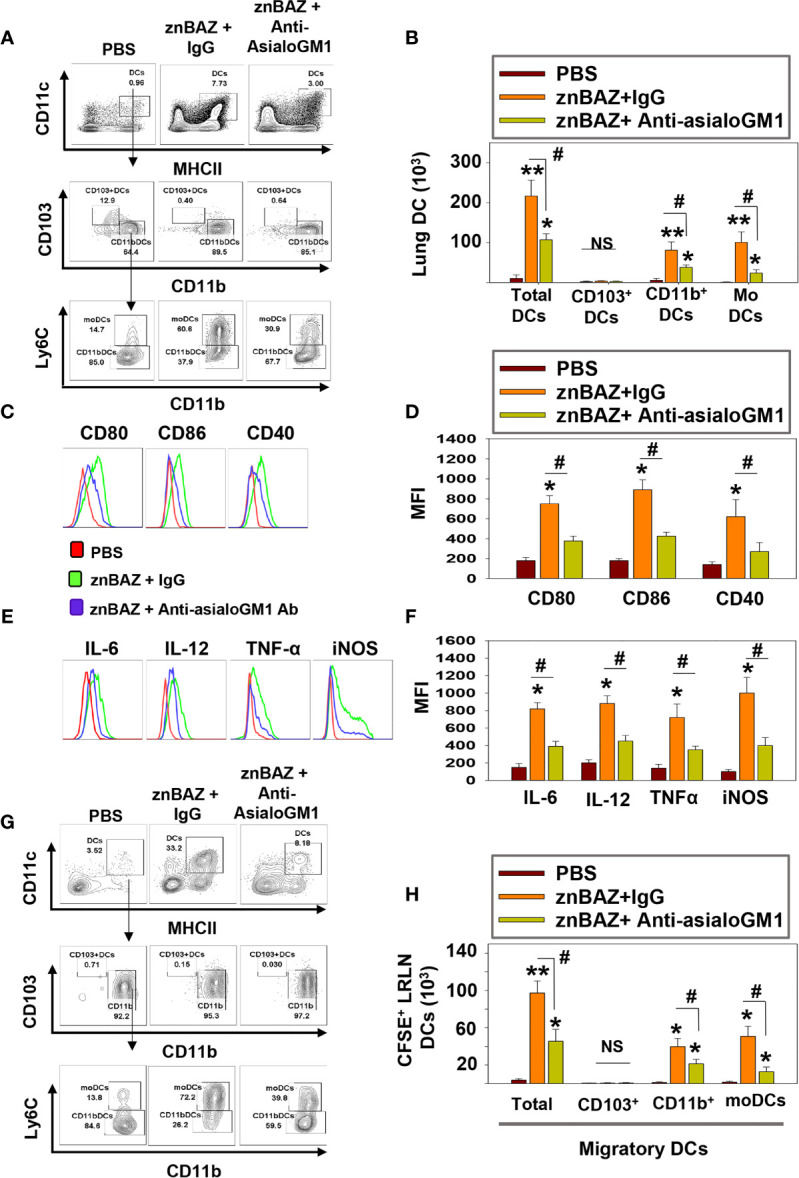
znBAZ induces early NK cell activation, and enhances lung DC maturation and migration to the lower respiratory lymph nodes (LRLNs). **(A–H)** Groups of BALB/c mice were nasally infected with znBAZ (1×10^9^ CFUs) on day 0. One half of znBAZ-infected mice were depleted of NK cells with rabbit anti-asialo-GM1 Ab, and the other half with an equivalent amount of normal rabbit IgG on days -1 and +2. Flow cytometry analysis was performed on day 5 post-infection to determine the **(A, B)** various DC subset numbers: total MHC class II^high^ CD11c^+^ DCs, CD103^+^ DCs, CD11b^+^ DCs and monocytic DCs (moDCs); **(C, D)** their activation status *via* expression of **(C)** CD80, CD86, and CD40 expression and **(D)** respective mean fluorescence intensity (MFI); and **(E)** their expression and **(F)** MFIs for IL-6, IL-12, TNF-α, and iNOS. **(G, H)** Anti-asialo-GM1 Ab treatment was performed as described above, and mouse lung mononuclear cells were labeled in *vivo* with CFSE 6 hrs before nasal znBAZ infection. On day 5 post-infection, flow cytometry analysis of migratory CFSE^+^ lung DCs in LRLNs was performed on individual mice from the three treatment groups, and the **(H)** specific numbers are shown. The data depict are the means ± SEM of 5 mice/group; *p < 0.01 and **p ≤ 0.001, ^#^ *p <* 0.01, compared with sPBS-dosed mice or as indicated; NS, not significant. Data are representative of two or three experiments.

DCs are involved in the initiation of adaptive immune responses, and are major envoys between innate and adaptive immune systems (39). Antigen uptake and presentation by DCs are critical for priming T cell responses. NK cells have been reported to interact with DCs and modulate their activation (40). Depending on their activation status and cytokine profiles, DCs induce distinct T cell polarization to shape the immune response (41). Under steady-state conditions, mouse lungs have two major DC subsets: CD103^+^ DC (CD11c^+^, MHCII^hi^, CD103^+^, CD11b^-^) and CD11b^+^ DC (CD11c^+^ MHCII^hi^ CD103^-^ CD11b^+^). During infection and inflammation, a third subset appears, referred to as monocytic DCs (moDC; CD11c^+^ MHCII^hi^ CD103^-^ CD11b^+^ Ly6C^+^) (41). We queried whether znBAZ-induced lung NK cells can modulate lung DC phenotypes. A gating strategy for lung DC analysis is provided in **Supplementary Figure 3**. To determine NK cells’ relevance following their depletion, groups of BALB/c mice were i.p. treated with either anti-asialo-GM1 or equivalent rabbit IgG control Ab one day prior to nasal znBAZ infection and two days thereafter. On day 5 post-infection, a significant reduction in total lung DCs was observed in NK cell-depleted mice compared to normal rabbit IgG-treated mice (**Figures 4A, B**). CD103^+^ DCs were absent from any of the treated groups (**Figure 4B**). Both CD11b^+^ DCs and moDCs were induced by znBAZ infection, and these were significantly reduced by 2.1- and 4.3-fold, respectively in NK cell-depleted mice (**Figure 4B**). Examination of lung DCs from wt *B. abortus* 2308 was unrevealing (data not shown), and results were similar to DC phenotypes previously described for *B. abortus*-infected lung DCs (42).

To assess the activation state of total lung DCs in znBAZ-infected mice with or without NK cells, expression levels of co-stimulatory molecules, CD40, CD80, and CD86, and inflammatory mediators, IL-6, IL-12p70, TNF-α, and iNOS were measured. DCs from NK cell-depleted mice exhibited a less activated phenotype compared to DCs from IgG-treated mice noted by reductions in CD80, CD86, and CD40 expression as well as reductions in their corresponding MFIs (**Figures 4C, D**). Examination of cytokine responses revealed reduction in intracellular IL-6, IL-12p70, and TNF-α levels, and MFIs subsequent anti-asialoGM1 Ab treatment (**Figures 4D, F**). Depleting NK cells also reduced the DC iNOS levels (**Figures 4E, F**). Additional analysis was performed examining the effect of NK cell depletion on lung DC migration to LRLNs by CFSE labeling. Mice were nasally treated with CFSE 6 hr prior to znBAZ infection, and 5 days later, the CFSE^+^ cells in the LRLNs were analyzed for DCs. The gating strategy for migratory DC analysis is provided in **Supplementary Figure 4A**. Total CFSE^+^ DCs increased in the LRLNs following znBAZ infection relative to naïve (PBS) controls. The majority of the migratory DCs in the LRLNs were split between CD11b^+^ DCs and moDCs (**Figures 4G, H**). In those znBAZ-infected mice treated with the anti-asialoGM1 Ab (**Figure 4H**), no changes were observed for CD103^+^ DCs, but CD11b^+^ DCs and moDCs increased in the LRLNs subsequent to znBAZ infection. In NK cell-depleted mice, both CD11b^+^ DCs and moDCs were reduced by 2- and 4-fold, respectively (**Figures 4G, H**). Together, these observations underscore an essential role for lung NK cells in lung DC activation and migration to the LRLNs.

### znBAZ Infection Induces IFN-γ-Dependent Lung DC Maturation and Migration

**Figure 5:**
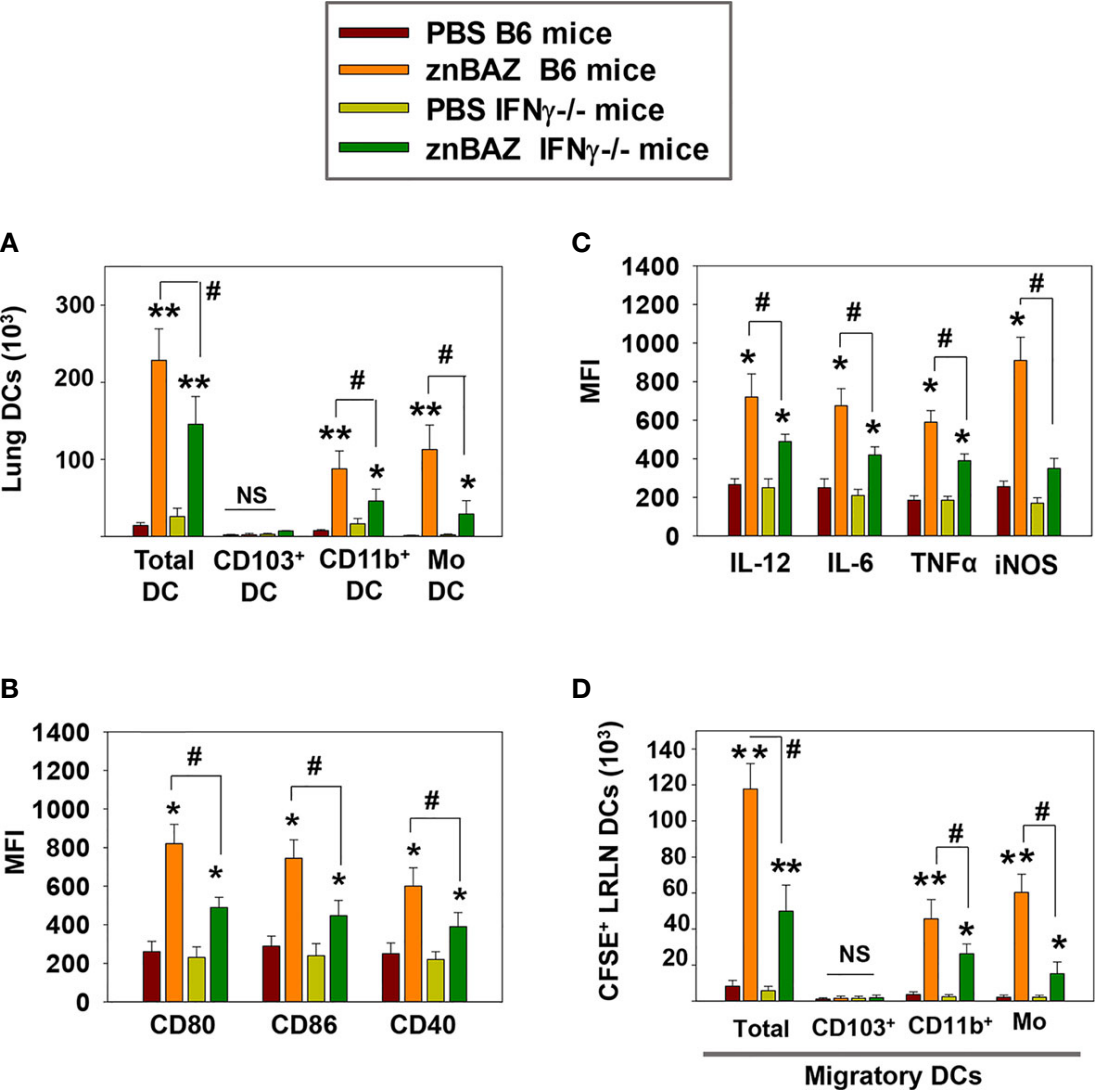
IFN-γ plays a key role in NK cell-mediated lung DC maturation and migration following znBAZ infection. Groups of B6 and IFN-γ^-/-^ mice were nasally infected with znBAZ (1×10^9^ CFUs). **(A–C)** Flow cytometry analysis was performed on day 5 post-infection to quantify the numbers of lung DC subsets: **(A)** total lung MHC class II^high^ CD11c^+^ DCs, CD103^+^ DCs, CD11b^+^ DCs and monocytic DCs (moDCs); and **(B)** MFI for their expression of CD80, CD86, CD40 and **(C)** MFI for IL-12, IL-6, TNF-α, and iNOS. **(D)** To measure the number of migratory CFSE^+^ lung DCs to the LRLNs, flow cytometry analysis was also performed on day 5 post-znBAZ infection of B6 and IFN-γ^-/-^ mice. The data depict the means ± SEM of 5 mice/group; *p < 0.01 and **p ≤ 0.001, compared with PBS-dosed mice, ^#^ *p <* 0.01 compared to znBAZ B6 mice; NS, not significant. Data are representative of two experiments.

IFN-γ secreted from activated NK cells regulates T cell priming either directly or by modulating the maturation and migration of DCs. To determine the role of NK cell secreted IFN-γ in lung DC activation and migration upon znBAZ infection, B6 and IFN-γ^-/-^ mice were nasally infected with znBAZ, and on day 5 post-infection, the lung DC subsets were analyzed. Fewer total lung DCs were observed in znBAZ-infected IFN-γ^-/-^ mice compared to those from similarly infected B6 mice (**Figure 5A**). While no CD103^+^ DCs were obtained for either treatment group or species, the CD11b^+^ DCs and moDCs were significantly induced in IFN-γ^-/-^ mice, but not to the degree as observed with the B6 mice. In fact, the moDCs was reduced 3.8-fold, the CD11b^+^ DCs, 1.9-fold (**Figure 5A**). The lung DC activation status was also significantly compromised in IFN-γ^-/-^ mice, evidenced by the reduction in MFIs for CD80, CD86, and CD40 (**Figure 5B**). In a similar fashion, DC-derived IL-6, IL-12p70, TNF-α, and iNOS were significantly reduced in znBAZ-infected IFN-γ^-/-^ mice (**Figure 5C**). Using the same *in vivo* labeling method as was applied for **Figure 3H**, the IFN-γ^-/-^ DCs were not as effective in recruiting pulmonary DCs to the LRLNs following znBAZ infection. DC recruitment to LRLNs was significantly reduced in IFN-γ^-/-^ mice (**Figure 5D**). The reductions impacted both CD11b^+^ DCs and moDCs by 1.7-fold and 3.9-fold, respectively, relative to those obtained in B6 mice. These data point to the relevance of IFN-γ produced by lung NK cells mediating lung DC activation and migration to LRLNs during the early course of vaccination.

### Early NK Cell Response Is Crucial for Lung CD8^+^ T Cell-Priming

**Figure 6:**
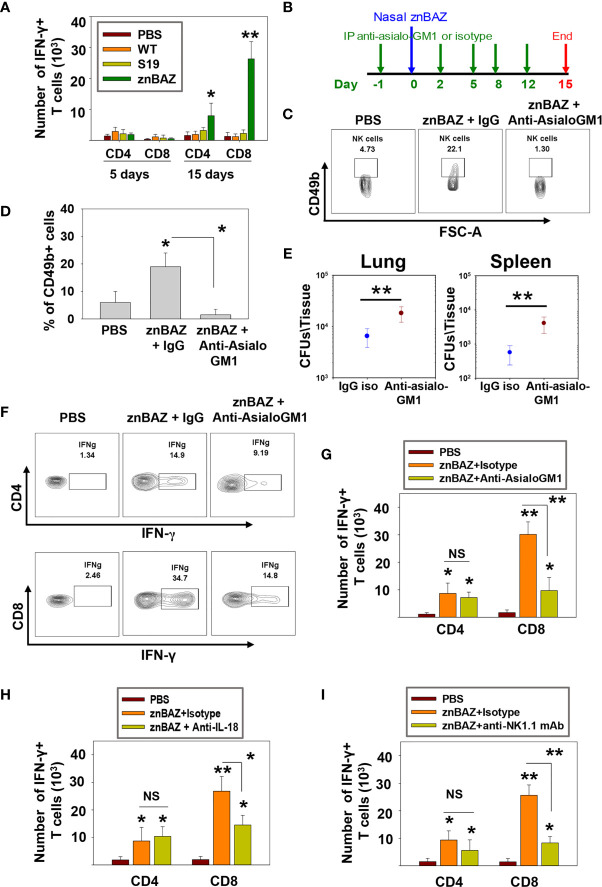
znBAZ induces an early NK cell response that augments CD8^+^ T cell response in the lungs. **(A)** Lung lymphocytes were analyzed on days 5 and 15 post-infection for IFN-γ-producing CD4^+^ and CD8^+^ T cells from BALB/c mice nasally infected with wt *B. abortus* (1×10^5^ CFUs), S19 (1×10^8^ CFUs), or znBAZ (1×10^9^ CFUs). **(B)** Groups of BALB/c mice were nasally infected with znBAZ (1×10^9^ CFUs) or sPBS on day 0. One half of znBAZ-dosed mice was depleted of NK cells with rabbit anti-asialo-GM1 Ab, and the other half was treated with an equivalent amount of normal rabbit IgG on days -1, 2, 5, 8, and 12. **(B, C)** NK cell depletion in the lungs was verified on days 5 and **(D)** 15 post-infection. On day 15 post-infection, **(E)** lungs and spleen from mice dosed with znBAZ + IgG or znBAZ + anti-asialo-GM1 Ab were evaluated for extent of znBAZ colonization, and **(F, G)** for the number of IFN-γ-producing CD4^+^ and CD8^+^ T cells. **(H)** Separate groups of BALB/c mice were nasally dosed with znBAZ (1×10^9^ CFUs) or sPBS on day 0. One half of znBAZ-dosed mice were treated i.p. with an anti-IL-18 mAb and the other half with an equivalent amount IgG isotype control on days -1, 2, 5, 8, and 12. Analysis of lung lymphocytes for IFN-γ-producing CD4^+^ and CD8^+^ T cells was performed on day15 post-infection. **(I)** Groups of B6 mice were treated with anti-NK1.1 mAb to deplete NK cells or IgG isotype control Ab, and dosed with znBAZ as described in **(B)**. sPBS was administered to one group as negative control. Analysis of lung IFN-γ-producing CD4^+^ and CD8^+^ T cells in control and NK cell-depleted B6 mice is shown at 15 days post-infection. The data depict the means ± SEM of 10 mice/group (two experiments combined); *p < 0.01 and **p ≤ 0.001, compared with sPBS-dosed mice or as indicated; NS is not significant.

Data thus far show that lung NK cells act upon lung APCs influencing their activation status and cytokine production levels. Such interactions are conducive in driving IFN-γ-dependent responses. Hence, we queried whether lung NK cells can influence pulmonary T cell responses. Groups of BALB/c mice nasally dosed with sPBS, wt *B. abortus* 2308, S19, or znBAZ. At 5 and 15 days after infection, lungs were examined for numbers of CD4^+^ and CD8^+^ T cells. Beginning at 5 days post-infection, no obvious change in IFN-γ-producing CD4^+^ and CD8^+^ T cells was noted. On day 15, the number of IFN-γ-producing CD4^+^ and CD8^+^ T cells in the lungs were significantly augmented in mice infected with znBAZ by 4- and 14-fold, respectively (**Figure 6A**). Conversely, neither wt *B. abortus*- or S19-infected mice showed any appreciable change in their lung IFN-γ-producing CD4^+^ or CD8^+^ T cell numbers relative to naïve mice.

To determine the impact of NK cell depletion in znBAZ-induced T cell responses, groups of mice were treated with the anti-asialo-GM1 Ab, and compared to BALB/c mice treated with rabbit IgG control Ab one day prior to nasal znBAZ infection, and every 3 days thereafter (**Figure 6B**). The NK cell depletion in the lungs was verified on day 5 post-infection (**Figures 6C, D**). Fifteen days after znBAZ infection, mice lungs were analyzed for znBAZ colonization of lungs and spleen and IFN-γ-producing CD4^+^ and CD8^+^ T cell levels. In the NK cell-depleted mice, significantly more brucellae were harbored in lungs and spleen compared to IgG-treated mice (**Figure 6E**), implicating that the loss of NK cells compromises znBAZ’s clearance. A 3.1-fold reduction in the CD8^+^ T cells was obtained when compared to IgG control-treated mice (**Figures 6F, G**). No changes in CD4^+^ T cell levels were observed in NK cell-depleted mice. To assess the impact of the reduced early lung NK cell activation on znBAZ-induced T cell response, the lung cells isolated from znBAZ infected BALB/c mice, treated with anti-IL-18 mAb or IgG isotype Ab, were analyzed on day 15 post-infection. A significant decline by 1.8-fold in the frequency of IFN-γ-producing lung CD8^+^ T cells in the znBAZ-vaccinated group was noted upon IL-18 neutralization (**Figure 6H**). To assess whether similar change occurs for CD8^+^ T cells in B6 mice, groups of mice were treated with an anti-NK1.1 mAb or its isotype control Ab. The NK cell depletion in B6 mice lungs was verified on day 5 post-infection (**Supplementary Figure 4B**). A significant 3-fold decrease in znBAZ-induced CD8^+^ T cells was observed in NK1.1-depleted mice, along with no appreciable difference in CD4^+^ T cells (**Figure 6I**). It has been reported that infection with some pathogens induces asialo-GM1^+^ CD8^+^ T cells in BALB/c mice and NK1.1^+^ CD8^+^ T cells in C57BL/6 mice (43, 44). However, in nasally znBAZ-infected mice, no lung asialo-GM1^+^ nor NK1.1^+^ CD8^+^ T cells were observed (**Supplementary Figures 5A, B**). Hence, depletion of NK cells using anti-asialo-GM1 Ab or anti-NK1.1 mAb was not directly responsible for lysing CD8^+^ T cells, similar to that found by others (45).

**Figure 7:**
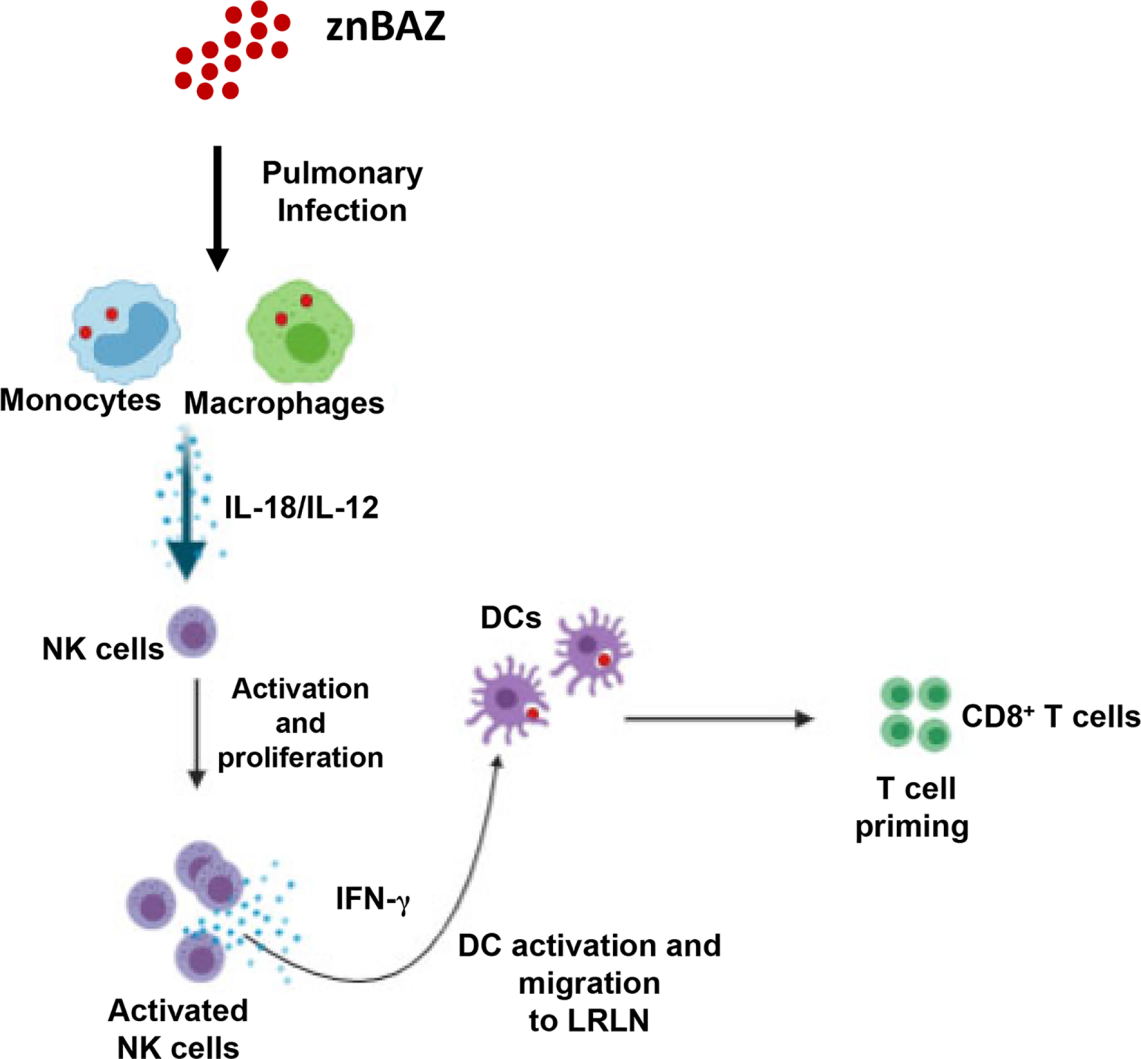
A schematic representation * highlighting the role NK cells in znBAZ-induced CD8^+^ T cell response. znBAZ is phagocytosed by lung inflammatory monocytes and monocyte-derived macrophages, which upon activation releases IL-12 and IL-18. These cytokines induce early NK cell activation. Early activation of lung NK cells releases granzyme B and IFN-γ. Granzyme B induces death (apoptosis/necrosis or both) of infected cells, and IFN-γ induces lung DC activation. These activated lung DCs uptake the bacterial Ags or engulf znBAZ or indirectly by phagocytizing infected host cells. After Ag uptake, DCs then migrate to lower respiratory lymph nodes (LRLNs), where they prime naïve T cells. These DC-primed CD8^+^ T cells becomes activated, undergo differentiation, and migrate to the site of infection (lungs) to provide protection. (Depiction was used generated using a program from BioRender.com).

Collectively, these results demonstrate that NK cells are essential for priming IFN-γ-producing CD8^+^ T cells in mice nasally dosed with znBAZ (**Figure 7**). Our data suggest that pulmonary NK cells are activated by IL-18 and IL-12 produced by znBAZ-infected MDMs and monocytes. The activated NK cells in turn co-activate infected or *Brucella* Ag-bearing CD11b^+^ DCs and moDCs *via* IFN-γ to drive the stimulation of *Brucella* Ag-responsive CD8^+^ T cells.

## Discussion

Although mucosal exposure is the most common route of *Brucella* infection, parenteral vaccination is still the route of choice for live vaccine administration (46). It is well established that mucosal vaccination elicits both local and systemic immunity (47). Therefore, adopting a mucosal vaccination should be considered as an alternate strategy to protect against natural *Brucella* exposure.

This study shows that nasal vaccination with a live, attenuated *B. abortus* znBAZ mutant induces an early NK cell response in the lungs in both BALB/c and C57BL/6 mice. NK cells have an important role in generating host resistance to various bacterial, fungal, viral, and parasitic infections at mucosal tissues by coordinating innate and adaptive immune responses (48, 49). Studies were performed in both mouse strains to allay concerns regarding Th cell bias. In fact, equivalent full protection was achieved upon znBAZ vaccination of either mouse strain, and Th1 cell bias associated with B6 mice did not influence efficacy (31). For both mouse strains, znBAZ infection increases lung NK cells’ frequency between 2 to 5 days post-infection and decreases thereafter. This early surge in lung NK cells is attributed to both their recruitment and proliferation. NK cell recruitment is primarily mediated by chemokine receptor, CCR2, CCR5, and CXCR3 signaling (50). Analysis of lung NK cells two days post-infection reveals that these are CXCR3^+^ and exhibit the Ki67 proliferation marker, suggesting that znBAZ infection induces early NK cell infiltration and then, proliferation. NK cells are also an important early source of IFN-γ used to modulate adaptive immune responses, particularly by driving Th1 cell polarization (51). As demonstrated here, znBAZ induces NK cells to produce a significant amount of IFN-γ. These NK cells also show an increased activation status for NKp46 and granzyme B, and reduced NK cell inhibitory molecule, NKG2A. In contrast, nasal infection with either wt *B. abortus* 2308 or with the S19 vaccine failed to elicit the early NK cell response in murine lungs. These latter findings are consistent with that previously reported in that NK cells had minimal to no impact upon infection with wt *B. abortus* 2308 (52). In contrast, brucellae lung colonization with znBAZ was significantly enhanced in the lungs and spleen by NK cell depletion. Hence, a significant finding is that znBAZ augments NK cell numbers and behaves differently from wt *Brucella*.

In peripheral tissues, the interaction between myeloid cells and NK cells is a major first-line defense against pathogenic infections (25). Recruitment and NK cells’ activation at the site of infection requires chemokines (ligands for CCR2, CCR5, and CXCR3) and cytokines (IL-12 and IL-18) secreted from myeloid cells, mainly monocytes and macrophages. It has been reported that lung resident macrophages provide a replicative niche for wt *Brucella* strains (42). Within alveolar macrophages, *Brucella* inhibits host cell apoptosis, evades immune surveillance which in turn makes more difficult for antigen processing and presentation (53, 54). In addition, during pulmonary infection with wt *Brucella*, alveolar macrophages inhibit pulmonary DC activation. Depletion of lung macrophages led to greater brucellae uptake by lung DCs, and induced a stronger inflammatory response (42). In contrast to these findings, nasal znBAZ infection reduced resident alveolar and interstitial macrophage populations and strongly induced inflammatory monocytes and MDMs into the lungs supporting the notion that the stealth traits associated with wt *Brucella* are lessened by the introduction of the genetic mutations in znBAZ. Analysis of BAL fluid revealed elevated IL-12 and IL-18, as well as CXCR3 ligands, CXCL-9 and CXCL-10, but not in BAL fluids from wt *B. abortus*- or S19-infected mice. Flow cytometry analysis reveals that the znBAZ-induced lung monocytes and MDMs act as sources for these chemokines and cytokines. Moreover, these cells were also FLICA positive, an indicator of caspase-1 activation, required for active IL-18 secretion. The synergistic action of IL-12 and IL-18 in the activation and IFN-γ secretion by NK cells is well-established (37). IL-18’s relevance in znBAZ-induced responses is particularly highlighted in having a major role in mediating NK cell activation. In fact, IL-18-deficient NK cells were unable to secrete IFN-γ in response to IL-12 stimulation (37, 38, 55). Therefore, the contribution of znBAZ-induced IL-18 for NK cell IFN-γ production was examined. *In vivo* IL-18 neutralization led to a significant reduction in znBAZ-induced lung NK cell frequency and IFN-γ production.

NK cell-derived IFN-γ plays a key role in modulating DC function at the site of infection (56). In line with these studies, our data provide evidence that znBAZ induced early lung NK cells supporting DCs activation and migration to LRLNs. These CD11b^+^ DCs and moDCs from znBAZ-infected lungs also served as a source of IL-6, IL-12, and TNF-α, and their activation were significantly abrogated upon NK cell depletion. Likewise, experimental analysis in B6 and IFN-γ^-/-^ mice revealed that IFN-γ plays a key role in the NK cell-mediated DC activation. NK cells also contributed to DC migration to draining lung LNs noted by reduced DC migration in the absence of NK cells or in the absence of IFN-γ. Hence, NK cells have an active role in the stimulation of APCs and ultimately the APCs’ migration to initiate and establish T cell responses.

Past studies have shown the importance of IL-18-primed NK cells to promote recruitment and activation of effector CD8^+^ T cells *via* DC activation and migration (57, 58). This current study shows that mice nasally dosed once with znBAZ develop a significant increase in the number of IFN-γ^+^ CD4^+^ and CD8^+^ T cells in the lungs. The CD8^+^ T cell response is remarkably elevated compared to CD4^+^ T cell response. Similar infection with wt *B. abortus* 2308 and S19 failed to induce any significant T cell response in the lungs, even when administered *via* the nasal route. Such evidence suggests that stimulation of CD8^+^ T cells is dependent on the *Brucella* strain, not the route of administration. Previous work showed that mucosal znBAZ vaccination protects against pulmonary wt *B. abortus* challenge, and this protection is CD8^+^ T cell-dependent (31). To verify the role of znBAZ-induced NK cells upon T cell responses, NK cell depletion was conducted in BALB/c and B6 mice. In both mouse strains, a significant decrease in znBAZ-induced IFN-γ^+^ CD8^+^ T cells was observed, and the CD4^+^ T cell response remained unaffected. As shown, IL-18 is critical for znBAZ-induced lung NK cell activation. Both NK cell depletion and *in vivo* IL-18 neutralization studies demonstrated these negatively impacted the stimulation of CD8^+^ T cell responses.

Collectively, these data show that nasal znBAZ vaccination induces chemokines and cytokines from lung monocytes and MDMs that are responsible for NK cell recruitment and activation in mouse lungs. Upon activation, these NK cells secrete IFN-γ, which in turn modulates lung DC maturation and migration to LRLNs. The early NK cell activation in the lungs is ultimately important for znBAZ-induced CD8^+^ T cell responses in the lungs. These findings provide mechanistic details of how znBAZ stimulates innate lymphocytes to support CD8^+^ T cell responses. These findings can be further used to aid in the design for improved mucosal vaccines against *Brucella* sp.

## Data Availability Statement

The original contributions presented in the study are included in the article/**Supplementary Material**. Further inquiries can be directed to the corresponding author.

## Ethics Statement

The animal study was reviewed and approved by University of Florida Institutional Animal Care and Use Committee.

## Author Contributions

Conceptualization: EB, HW, and DP. Formal analysis: EB, HW, AA, and DP. Funding acquisition: DP. Investigation: EB, HW, XY, CH, AA, ZG, and DP. Methodology: EB, HW, XY, CH, AA, ZG, and DP. Validation: EB, HW, XY, CH, AA, ZG, and DP. Visualization: EB, HW, CH, AA, and DP. Writing: EB, HW, XY, AA, ZG, and DP. All authors contributed to the article and approved the submitted version.

## Funding

This work was supported by National Institute of Allergy and Infectious Diseases grants AI123244 and AI125516, and NIH 1S10 OD021676 (DP).

## Conflict of Interest

The authors declare that the research was conducted in the absence of any commercial or financial relationships that could be construed as a potential conflict of interest.

## References

[1] Pappas G Papadimitriou P Akritidis N Christou L Tsianos EV. The New Global Map of Human Brucellosis. Lancet Infect Dis (2006) 6(2):91–9. 10.1016/S1473-3099(06)70382-6 16439329

[2] Zhou K Wu B Pan H Paudyal N Jiang J Zhang L. ONE Health Approach to Address Zoonotic Brucellosis: A Spatiotemporal Associations Study Between Animals And Humans. Front Vet Sci (2020) 7:521. 10.3389/fvets.2020.00521 32984409 PMC7492289

[3] Olsen SC Palmer MV. Advancement of Knowledge of *Brucella* Over the Past 50 Years. Vet Pathol (2014) 51(6):1076–89. 10.1177/0300985814540545 24981716

[4] Pappas G Akritidis N Bosilkovski M Tsianos E. Brucellosi. N Engl J Med (2005) 352(22):2325–36. 10.1056/NEJMra050570 15930423

[5] Khan MZ Zahoor M. An Overview of Brucellosis in Cattle and Humans, and its Serological and Molecular Diagnosis in Control Strategie. Trop Med Infect Dis (2018) 3(2):65. 10.3390/tropicalmed3020065 PMC6073575 30274461

[6] Kaufmann AF Fox MD Boyce JM Anderson DC Potter ME Martone WJ. Airborne Spread of Brucellosis. Ann N Y Acad Sci (1980) 353:105–14. 10.1111/j.1749-6632.1980.tb18912.x 6939379

[7] Moreno E. Retrospective and Prospective Perspectives on Zoonotic Brucellosis. Front Microbiol (2014) 5:213. 10.3389/fmicb.2014.00213 24860561 PMC4026726

[8] Hull NC Schumaker BA. Comparisons of Brucellosis Between Human and Veterinary Medicine. Infect Ecol Epidemiol (2018) 8(1):1500846. 10.1080/20008686.2018.1500846 30083304 PMC6063340

[9] Dean AS Crump L Greter H Schelling E Zinsstag J. Global Burden of Human Brucellosis: A Systematic Review of Disease Frequency. PloS Negl Trop Dis (2012) 6(10):e1865. 10.1371/journal.pntd.0001865 23145195 PMC3493380

[10] Franc KA Krecek RC Hasler BN Arenas-Gamboa AM. Brucellosis Remains a Neglected Disease in the Developing World: A Call for Interdisciplinary Action. BMC Public Health (2018) 18(1):125. 10.1186/s12889-017-5016-y 29325516 PMC5765637

[11] Galinska EM Zagorski J. Brucellosis in Humans–Etiology, Diagnostics, Clinical Forms. Ann Agric Environ Med (2013) 20(2):233–8. 23772567

[12] Johansen MV Welburn SC Dorny P Brattig NW. Control of Neglected Zoonotic Diseases. Acta Trop (2017) 165:1–2. 10.1016/j.actatropica.2016.11.036 27964807

[13] de Figueiredo P Ficht TA Rice-Ficht A Rossetti CA Adams LG. Pathogenesis and Immunobiology of Brucellosis: Review of *Brucella*-Host Interactions. Am J Pathol (2015) 185(6):1505–17. 10.1016/j.ajpath.2015.03.003 PMC4450313 25892682

[14] Dean AS Crump L Greter H Hattendorf J Schelling E Zinsstag J. Clinical Manifestations of Human Brucellosis: A Systematic Review and Meta-Analysis. PloS Negl Trop Dis (2012) 6(12):e1929. 10.1371/journal.pntd.0001929 23236528 PMC3516581

[15] Demars A Lison A Machelart A Van Vyve M Potemberg G Vanderwinden JM. Route of Infection Strongly Impacts the Host-Pathogen Relationship. Front Immunol (2019) 10:1589. 10.3389/fimmu.2019.01589 31354728 PMC6637429

[16] Neutra MR Kozlowski PA. Mucosal Vaccines: The Promise and the Challenge. Nat Rev Immunol (2006) 6(2):148–58. 10.1038/nri1777 16491139

[17] Pavot V Rochereau N Genin C Verrier B Paul S. New Insights in Mucosal Vaccine Development. Vaccine (2012) 30(2):142–54. 10.1016/j.vaccine.2011.11.003 22085556

[18] Kim ED Han SJ Byun YH Yoon SC Choi KS Seong BL. Inactivated Eyedrop Influenza Vaccine Adjuvanted With Poly(I:C) Is Safe and Effective for Inducing Protective Systemic and Mucosal Immunit. PloS One (2015) 10(9):e0137608. 10.1371/journal.pone.0137608 26355295 PMC4565664

[19] Higgs R Higgins SC Ross PJ Mills KH. Immunity to the Respiratory Pathogen *Bordetella pertussis* . Mucosal Immunol (2012) 5(5):485–500. 10.1038/mi.2012.54 22718262

[20] Iwasaki A Foxman EF Molony RD. Early Local Immune Defences in the Respiratory Tract. Nat Rev Immunol (2017) 17(1):7–20. 10.1038/nri.2016.117 27890913 PMC5480291

[21] Sun JC Lanier LL. Natural Killer Cells Remember: An Evolutionary Bridge Between Innate and Adaptive Immunity? Eur J Immunol (2009) 39(8):2059–64. 10.1002/eji.200939435 PMC2819266 19637199

[22] Moretta A Marcenaro E Parolini S Ferlazzo G Moretta L. NK Cells at the Interface Between Innate and Adaptive Immunity. Cell Death Differ (2008) 15(2):226–33. 10.1038/sj.cdd.4402170 17541426

[23] Cong J Wei H. Natural Killer Cells in the Lung. Front Immunol (2019) 10:1416. 10.3389/fimmu.2019.01416 31293580 PMC6603080

[24] Ivanova D Krempels R Ryfe J Weitzman K Stephenson D Gigley JP. NK Cells in Mucosal Defense Against Infection. BioMed Res Int (2014) 2014:413982. 10.1155/2014/413982 25197644 PMC4150440

[25] Lodoen MB Lanier LL. Natural Killer Cells as an Initial Defense Against Pathogens. Curr Opin Immunol (2006) 18(4):391–8. 10.1016/j.coi.2006.05.002 PMC7127478 16765573

[26] Freeman BE Raue HP Hill AB Slifka MK. Cytokine-Mediated Activation of NK Cells During Viral Infection. J Virol (2015) 89(15):7922–31. 10.1128/JVI.00199-15 PMC4505636 25995253

[27] Zwirner NW Domaica CI. Cytokine Regulation of Natural Killer Cell Effector Functions. Biofactors (2010) 36(4):274–88. 10.1002/biof.107 20623510

[28] Long EO Kim HS Liu D Peterson ME Rajagopalan S. Controlling Natural Killer Cell Responses: Integration of Signals for Activation and Inhibition. Annu Rev Immunol (2013) 31:227–58. 10.1146/annurev-immunol-020711-075005 PMC3868343 23516982

[29] O’Connor GM Hart OM Gardiner CM. Putting the Natural Killer Cell in Its Place. Immunology (2006) 117(1):1–10. 10.1111/j.1365-2567.2005.02256.x 16423035 PMC1782192

[30] Cook KD Waggoner SN Whitmire JK. NK Cells and Their Ability to Modulate T Cells During Virus Infections. Crit Rev Immunol (2014) 34(5):359–88. 10.1615/CritRevImmunol.2014010604 PMC4266186 25404045

[31] Wang H Hoffman C Yang X Clapp B Pascual DW. Targeting Resident Memory T Cell Immunity Culminates in Pulmonary and Systemic Protection Against *Brucella* Infection. PloS Pathog (2020) 16(1):e1008176. 10.1371/journal.ppat.1008176 31951645 PMC6968852

[32] Yang X Clapp B Thornburg T Hoffman C Pascual DW. Vaccination With a Δ*norD* Δ*znuA Brucella abortus* Mutant Confers Potent Protection Against Virulent Challenge. Vaccine (2016) 34(44):5290–7. 10.1016/j.vaccine.2016.09.004 PMC5053898 27639282

[33] Legge KL Braciale TJ. Accelerated Migration of Respiratory Dendritic Cells to the Regional Lymph Nodes Is Limited to the Early Phase of Pulmonary Infection. Immunity (2003) 18(2):265–77. 10.1016/S1074-7613(03)00023-2 12594953

[34] Gu Y Kuida K Tsutsui H Ku G Hsiao K Fleming MA. Activation of Interferon-γ Inducing Factor Mediated by Interleukin-1β Converting Enzyme. Science (1997) 275(5297):206–9. 10.1126/science.275.5297.206 8999548

[35] Ghayur T Banerjee S Hugunin M Butler D Herzog L Carter A. Caspase-1 Processes IFN-Gamma-Inducing Factor and Regulates LPS-Induced IFN-γ Production. Nature (1997) 386(6625):619–23. 10.1038/386619a0 9121587

[36] Yasuda K Nakanishi K Tsutsui H. Interleukin-18 in Health and Diseas. Int J Mol Sci (2019) 20(3):649. 10.3390/ijms20030649 PMC6387150 30717382

[37] Nakahira M Ahn HJ Park WR Gao P Tomura M Park CS. Synergy of IL-12 and IL-18 for IFN-Gamma Gene Expression: IL-12-Induced STAT4 Contributes to IFN-γ Promoter Activation by Up-Regulating the Binding Activity of IL-18-Induced Activator Protein 1. J Immunol (2002) 168(3):1146–53. 10.4049/jimmunol.168.3.1146 11801649

[38] Gosmann C Frazer IH Mattarollo SR Blumenthal A. IL-18, But Not IL-12, Induces Production of IFN-Gamma in the Immunosuppressive Environment of HPV16 E7 Transgenic Hyperplastic Skin. J Invest Dermatol (2014) 134(10):2562–9. 10.1038/jid.2014.201 PMC4165718 24756108

[39] Steinman RM Hemmi H. Dendritic Cells: Translating Innate to Adaptive Immunity. Curr Top Microbiol Immunol (2006) 311:17–58. 10.1007/3-540-32636-7_2 17048704

[40] Ferlazzo G Morandi B. Cross-Talks Between Natural Killer Cells and Distinct Subsets of Dendritic Cell. Front Immunol (2014) 5:159. 10.3389/fimmu.2014.00159 24782864 PMC3989561

[41] Feili-Hariri M Falkner DH Morel PA. Polarization of Naive T Cells Into Th1 or Th2 by Distinct Cytokine-Driven Murine Dendritic Cell Populations: Implications for Immunotherapy. J Leukoc Biol (2005) 78(3):656–64. 10.1189/jlb.1104631 15961574

[42] Archambaud C Salcedo SP Lelouard H Devilard E de Bovis B Van Rooijen N. Contrasting Roles of Macrophages and Dendritic Cells in Controlling Initial Pulmonary *Brucella* Infection. Eur J Immunol (2010) 40(12):3458–71. 10.1002/eji.201040497 21108467

[43] Kosaka A Wakita D Matsubara N Togashi Y Nishimura S Kitamura H. AsialoGM1+CD8+ Central Memory-Type T Cells in Unimmunized Mice as Novel Immunomodulator of IFN-γ-Dependent Type 1 Immunity. Int Immunol (2007) 19(3):249–56. 10.1093/intimm/dxl140 17229818

[44] Ruiz AL Soudja SM Deceneux C Lauvau G Marie JC. NK1.1+ CD8+ T Cells Escape TGF-β Control and Contribute to Early Microbial Pathogen Response. Nat Commun (2014) 5:5150. 10.1038/ncomms6150 25284210 PMC4836950

[45] van der Touw W Burrell B Lal G Bromberg JS. NK Cells are Required for Costimulatory Blockade Induced Tolerance to Vascularized Allografts. Transplantation (2012) 94(6):575–84. 10.1097/TP.0b013e318264d3c4 PMC3448808 22914174

[46] Yang X Skyberg JA Cao L Clapp B Thornburg T Pascual DW. Progress in *Brucella* Vaccine Development. Front Biol (Beijing) (2013) 8(1):60–77. 10.1007/s11515-012-1196-0 23730309 PMC3666581

[47] Nizard M Diniz MO Roussel H Tran T Ferreira LC Badoual C. Mucosal Vaccines: Novel Strategies and Applications for the Control of Pathogens and Tumors at Mucosal Sites. Hum Vaccin Immunother (2014) 10(8):2175–87. 10.4161/hv.29269 PMC4896761 25424921

[48] Reid-Yu SA Small CL Coombes BK. CD3(-)NK1.1(+) Cells Aid in the Early Induction of a Th1 Response to an Attaching and Effacing Enteric Pathogen. Eur J Immunol (2013) 43(10):2638–49. 10.1002/eji.201343435 23775576

[49] Hall LJ Murphy CT Hurley G Quinlan A Shanahan F Nally K. Natural Killer Cells Protect Against Mucosal and Systemic Infection With the Enteric Pathogen *Citrobacter rodentium* . Infect Immun (2013) 81(2):460–9. 10.1128/IAI.00953-12 PMC3553819 23208605

[50] Pak-Wittel MA Yang L Sojka DK Rivenbark JG Yokoyama WM. Interferon-Gamma Mediates Chemokine-Dependent Recruitment of Natural Killer Cells During Viral Infection. Proc Natl Acad Sci USA (2013) 110(1):E50–9. 10.1073/pnas.1220456110 PMC3538256 23248310

[51] Scharton TM Scott P. Natural Killer Cells are a Source of Interferon γ That Drives Differentiation of CD4+ T Cell Subsets and Induces Early Resistance to *Leishmania Major* in Mice. J Exp Med (1993) 178(2):567–77. 10.1084/jem.178.2.567 PMC2191131 8101861

[52] Fernandes DM Benson R Baldwin CL. Lack of a Role for Natural Killer Cells in Early Control of *Brucella abortus* 2308 Infections in Mice. Infect Immun (1995) 63(10):4029–33. 10.1128/iai.63.10.4029-4033.1995 PMC173566 7558315

[53] Ma Z Li R Hu R Deng X Xu Y Zheng W. *Brucella abortus* BspJ Is a Nucleomodulin That Inhibits Macrophage Apoptosis and Promotes Intracellular Survival of *Brucella* . Front Microbiol (2020) 11:599205. 10.3389/fmicb.2020.599205 33281799 PMC7688787

[54] Zhou D Zhi FJ Qi MZ Bai FR Zhang G Li JM. *Brucella* Induces Unfolded Protein Response and Inflammatory Response *via* GntR in Alveolar Macrophages. Oncotarget (2018) 9(4):5184–96. 10.18632/oncotarget.23706 PMC5797042 29435171

[55] Chaix J Tessmer MS Hoebe K Fuseri N Ryffel B Dalod M. Cutting Edge: Priming of NK Cells by IL-18. J Immunol (2008) 181(3):1627–31. 10.4049/jimmunol.181.3.1627 PMC5154249 18641298

[56] Goldszmid RS Caspar P Rivollier A White S Dzutsev A Hieny S. NK Cell-Derived Interferon-γ Orchestrates Cellular Dynamics and the Differentiation of Monocytes Into Dendritic Cells at the Site of Infection. Immunity (2012) 36(6):1047–59. 10.1016/j.immuni.2012.03.026 PMC3412151 22749354

[57] Clavijo-Salomon MA Salcedo R Roy S das Neves RX Dzutsev A Sales-Campos H. Human NK Cells Prime Inflammatory DC Precursors to Induce Tc17 Differentiation. Blood Adv (2020) 4(16):3990–4006. 10.1182/bloodadvances.2020002084 32841340 PMC7448590

[58] Wong JL Berk E Edwards RP Kalinski P. IL-18-Primed Helper NK Cells Collaborate With Dendritic Cells to Promote Recruitment of Effector CD8+ T Cells to the Tumor Microenvironment. Cancer Res (2013) 73(15):4653–62. 10.1158/0008-5472.CAN-12-4366 PMC3780558 23761327

