# Supplementary material for: Mucosal Vaccination Primes NK Cell-Dependent Development of CD8+ T Cells Against Pulmonary Brucella Infection
Source: Front Immunol. 2021 Jul 7;12:697953. doi: 10.3389/fimmu.2021.697953 (PMC8293993; doi:10.3389/fimmu.2021.697953)
Supplement: Supplementary file 1 [file DataSheet_1.pdf]

Supplementary Figure 1

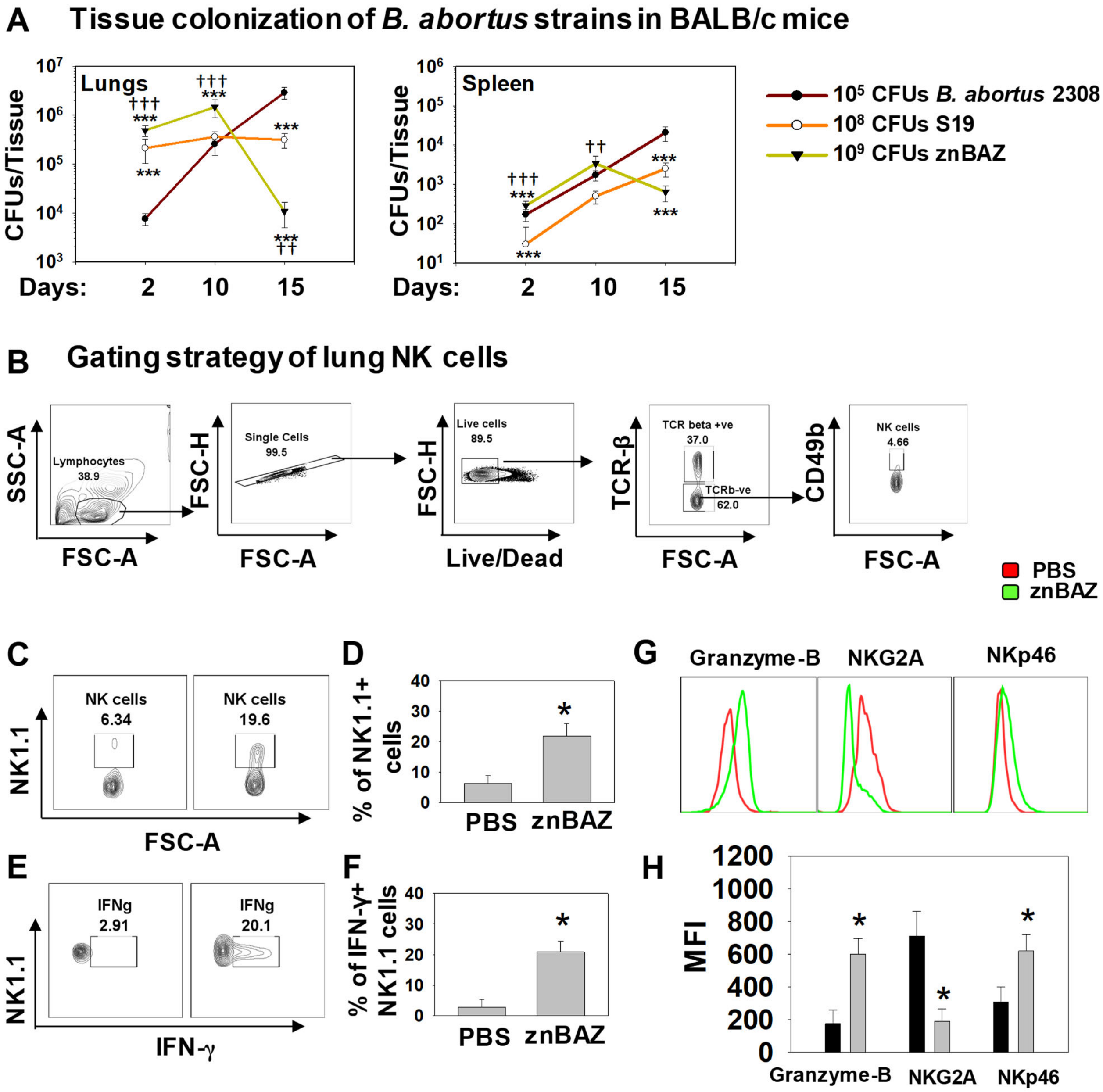

Figure S1: (A) Tissue colonization of *B. abortus* strains in BALB/c mice 2, 10, and 15 days after nasal infection; ***$p \leq 0.001$ vs wt BA 2308; $^{†††}p < 0.001$, $^{††}p \leq 0.014$ vs S19. (B) Representative flow cytometry gating strategy for lung NK cells in BALB/c mice. Flow cytometry analysis of lung NK1.1$^+$ (B,C) and IFN-γ producing NK1.1$^+$ NK cell (D,E) responses on day 5 post-znBAZ infection of C57BL/6 mice. (F,G) Expression analysis of granzyme B, NKG2A, and NKp46 by lung NK cells on day 5 post-znBAZ infection of C57BL/6 mice. The data depict

the means ± SEM of 5 mice/group; *$p < 0.01$ and **$p \leq 0.001$, compared with PBS-dosed mice or as indicated.

Supplementary Figure 2

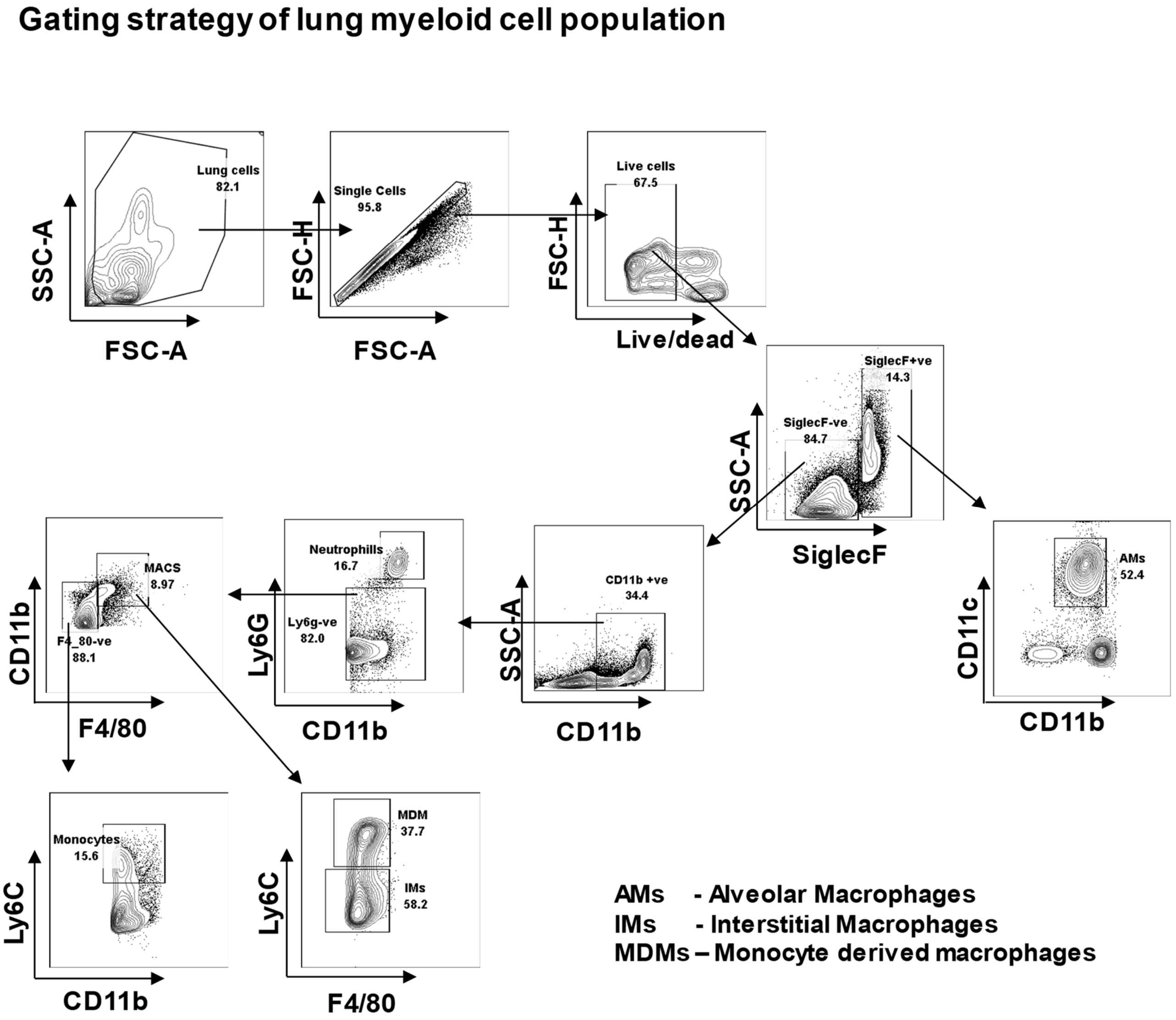

Figure S2: Representative flow cytometry gating strategy of lung myeloid cells: Alveolar macrophages (SiglecF^{+},CD11b^{lo},CD11c^{+}), Interstitial macrophages (SiglecF^{-},CD11b^{+}, Ly6G^{-}, F4/80^{+}, Ly6C^{-}), Monocyte derived macrophages (SiglecF^{-},CD11b^{+}, Ly6G^{-}, F4/80^{+}, Ly6C^{+}).

Supplementary Figure 3

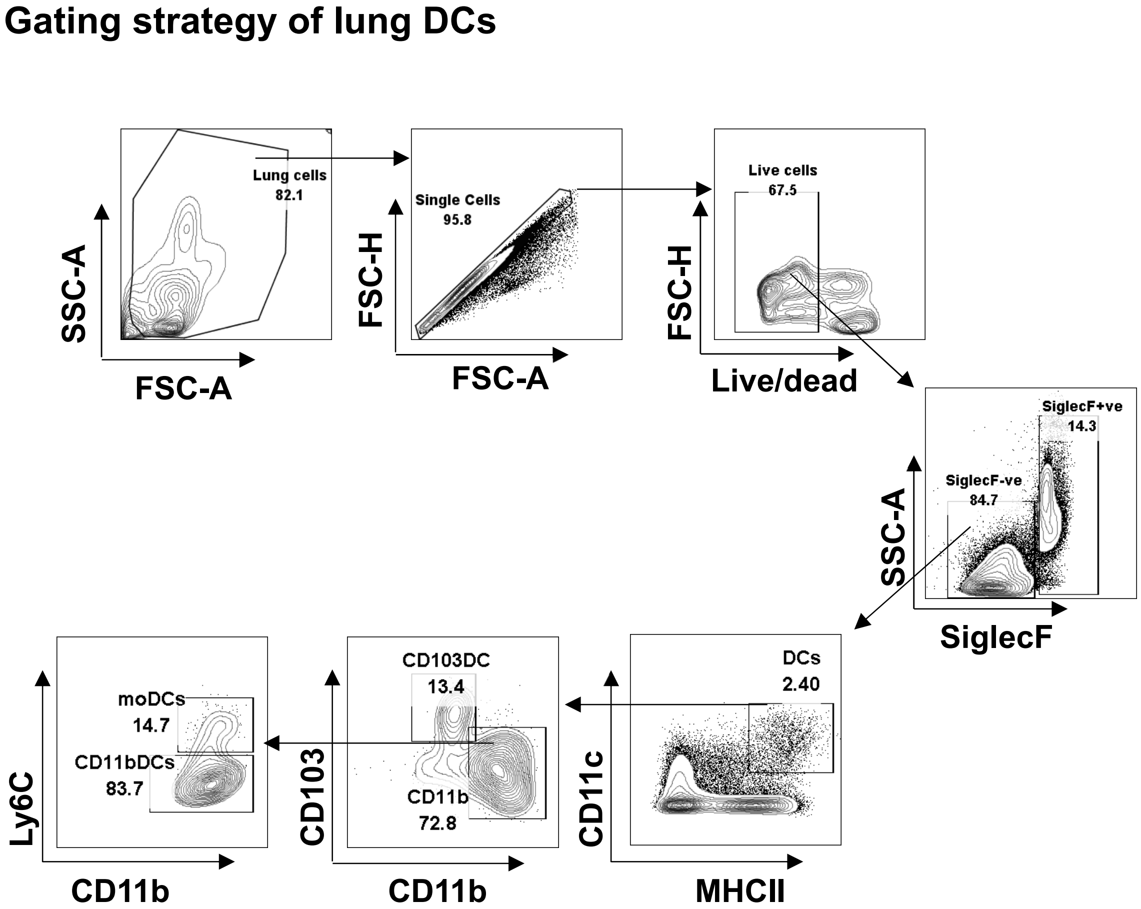

Figure S3: Representative flow cytometry gating strategy of lung dendritic cells (DCs): Total lung DCs (SiglecF^{-}, CD11c^{+}, MHCII^{hi}), CD103^{+} DCs (SiglecF^{-}, CD103^{+}, CD11c^{+}, MHCII^{hi}, CD11b^{-}), CD11b^{+} DCs (SiglecF^{-}, CD103^{-}, CD11c^{+}, MHCII^{hi}, CD11b^{+}, Ly6C^{-}), moDCs (SiglecF^{-}, CD103^{-}, CD11c^{+}, MHCII^{hi}, CD11b^{+}, Ly6C^{+})

Supplementary Figure 4

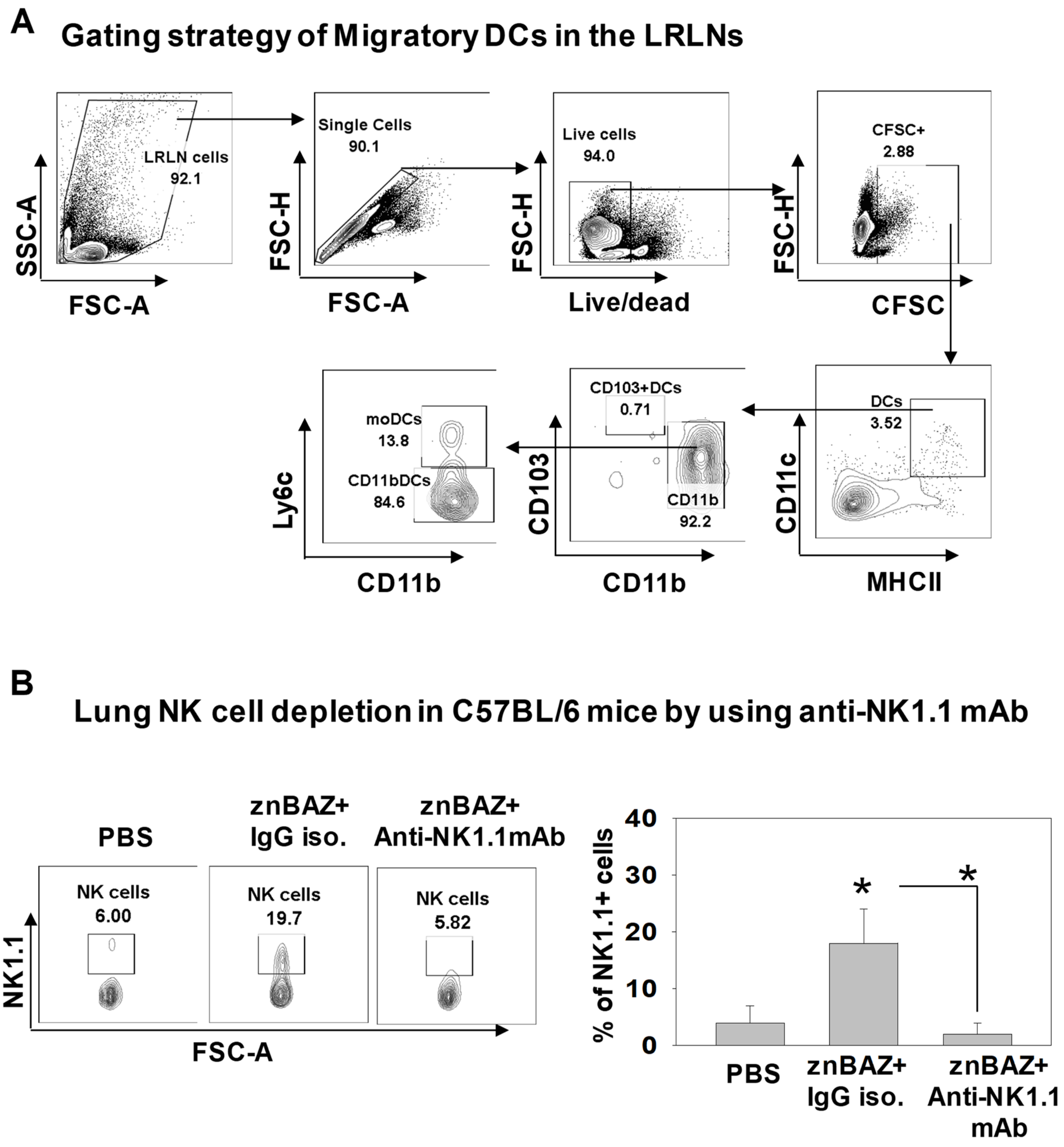

Figure S4: (A) Representative flow cytometry gating strategy of identifying lung migratory DCs into the LRLNs. (B) Analysis of lung NK cell depletion in znBAZ-infected C57BL/6 mice treated i.p with control IgG or anti-NK1.1 mAb. The data depict the means ± SEM of 5 mice/group; $^{*}p < 0.01$ and $^{**}p \leq 0.001$, compared with PBS-dosed mice or as indicated.

Supplementary Figure 5

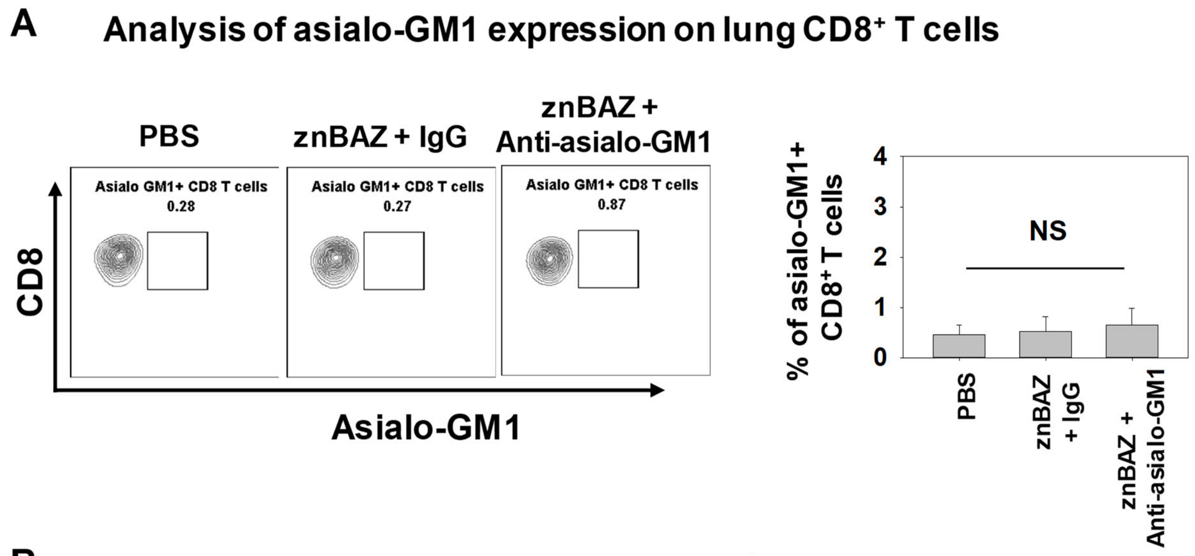

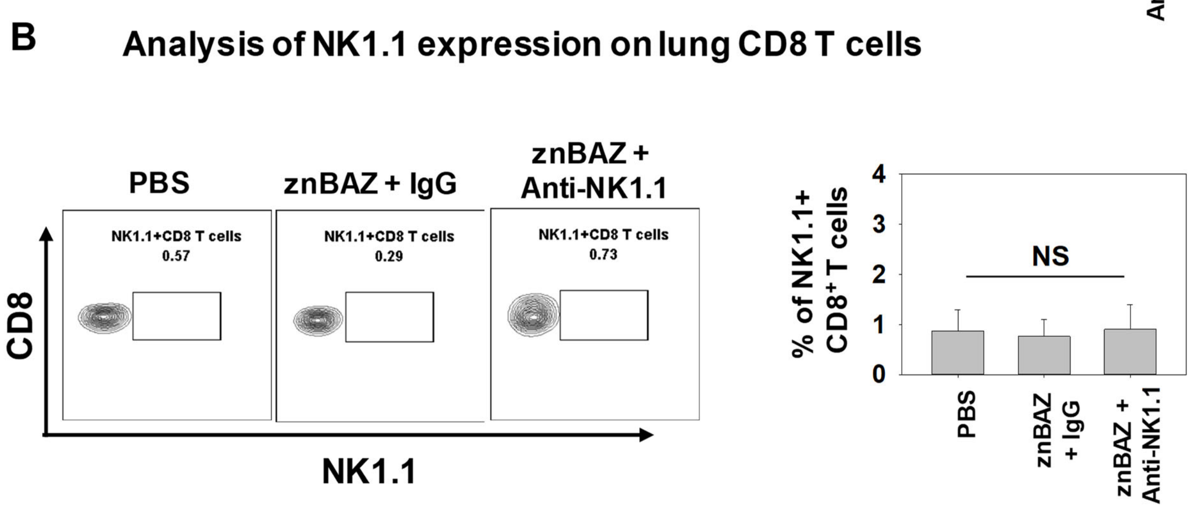

Figure S5: **(A)** Representative flow cytometry analysis of lung $CD8^{+}$ T cells showing asialo-GM1 positive $CD8^{+}$ T cells in znBAZ-infected BALB/c mice treated i.p with control IgG or asialo-GM1 Ab. **(B)** Representative flow cytometry analysis of lung $CD8^{+}$ T cells showing NK1.1 positive $CD8^{+}$ T cells in znBAZ-infected C57BL/6 mice treated i.p with control IgG or anti-NK1.1 mAb. The data depict the means ± SEM of 5 mice/group; NS (not significant).
